# Association Between the Early Postoperative Changes in Serum Brain Natriuretic Peptide and Allograft Survival After Kidney Transplantation: A Retrospective Cohort Study

**DOI:** 10.3390/jcm15082982

**Published:** 2026-04-14

**Authors:** Shih-Yu Chen, Chih-Chien Sung, Chien-Chang Kao, Sheng-Tang Wu, Wei-Hung Chan, Chun-Chang Yeh, Wei-Cheng Tseng

**Affiliations:** 1 Department of Anesthesiology, Tri-Service General Hospital, National Defense Medical University, Taipei 114, Taiwan; yooyooman33@gmail.com (S.-Y.C.); whcken@gmail.com (W.-H.C.); 2 Division of Nephrology, Department of Medicine, Tri-Service General Hospital, National Defense Medical University, Taipei 114, Taiwan; sungchihchien@gmail.com; 3 Division of Urology, Department of Surgery, Tri-Service General Hospital, National Defense Medical University, Taipei 114, Taiwan; guman2011@gmail.com (C.-C.K.); wushengtang89@gmail.com (S.-T.W.)

**Keywords:** allograft survival, brain natriuretic peptide, early graft function, end-stage kidney disease, kidney transplantation

## Abstract

**Background**: Kidney transplantation (KT) improves survival and quality of life in patients with end-stage kidney disease; however, long-term allograft survival remains a major challenge. Brain natriuretic peptide (BNP), a biomarker of cardiorenal stress and volume status, may be associated with early postoperative physiological changes after KT. This study evaluated the association between early postoperative BNP changes and long-term allograft survival, and explored the potential role of BNP-derived parameters in relation to graft outcomes. **Methods**: This retrospective cohort study included adult recipients of deceased-donor KT between 2009 and 2018. Patients were categorized according to early graft function. Serum BNP levels were measured preoperatively and within postoperative 24 h, and the percentage increase (dBNP ratio) was calculated. Cox regression and receiver operating characteristic analyses were used to identify risk factors for graft failure and evaluate the discriminatory performance of BNP-derived biomarkers, respectively. **Results**: Among the 179 recipients, postoperative BNP levels and dBNP ratios differed significantly across graft function groups, with higher values in delayed graft function. After multivariate adjustment, the dBNP ratio remained significantly associated with graft failure (hazard ratio, 1.16; 95% confidence interval, 1.10–1.21; *p* < 0.001). Additionally, the dBNP ratio demonstrated better discriminatory performance for graft failure compared with postoperative BNP alone (area under the curve, 0.815 vs. 0.596; *p* < 0.001), with an exploratory cutoff of approximately 18%. Recipients with a dBNP ratio ≥ 18% had poorer early graft function, lower longitudinal estimated glomerular filtration rates, and significantly reduced graft survival. **Conclusions**: An increased early postoperative dBNP ratio was significantly associated with adverse long-term kidney allograft outcomes. However, given the potential for residual confounding, these findings should be interpreted as associative and hypothesis-generating rather than predictive.

## 1. Introduction

Kidney transplantation (KT) is the treatment of choice for patients with end-stage kidney disease (ESKD), providing improved quality of life and superior survival compared with long-term dialysis [1]. However, allograft failure remains a significant clinical problem. Its causes are heterogeneous, multifactorial, and time-dependent. Within the first year after KT, allograft failure is primarily due to surgical issues, primary nonfunction and acute rejection; beyond one year, chronic alloimmune injury becomes the predominant cause [2]. Although advances in immunosuppressive therapy have markedly improved short-term graft survival [3,4], long-term outcomes remain suboptimal. The incidence of allograft loss, including death with a functioning graft, is approximately 5% per year beyond the first posttransplant year [4]. Therefore, early risk stratification and prediction of allograft failure are of critical importance.

Early allograft function following KT is a well-recognized determinant of long-term graft survival [5,6,7,8,9,10]. It is commonly categorized into immediate graft function (IGF), slow graft function (SGF), and delayed graft function (DGF). IGF is characterized by active diuresis and rapid decline in serum creatinine (sCr), whereas DGF typically requires dialysis within the first postoperative week [11]. SGF represents an intermediate phenotype, in which sCr does not decline immediately but dialysis is not required. However, the definition of SGF remains inconsistent, and its prognostic significance is controversial [8,9,10]. Importantly, these classifications are determined retrospectively, usually at postoperative day 7, which limits their utility for early clinical decision-making. Based on the above-mentioned reasons, there is a need for a simple and reliable biomarker associated with graft outcomes in the early posttransplant period.

Brain natriuretic peptide (BNP) is a polypeptide secreted primarily by cardiomyocytes in response to myocardial stretch and ischemia. It plays a key role in regulating fluid balance and cardiovascular homeostasis [12], and is widely used for diagnosis, risk stratification, and monitoring in heart failure [13,14]. Notably, in patients with ESKD, serum BNP levels can also reflect volume status [15,16]. Although the optimal cutoff for defining volume overload remains uncertain, a value of approximately 500 pg/mL has been proposed in dialysis populations [17]. Given the importance of fluid management after KT, BNP may serve as a surrogate marker of early postoperative volume status and may be associated with subsequent graft outcomes. However, the relationship between BNP levels and allograft survival after KT has not been well-established. Therefore, we conducted a single-center retrospective study to investigate the association between serum BNP levels and allograft survival and identify potential risk factors for graft loss.

## 2. Materials and Methods

### 2.1. Study Population and Data Collection

**Figure 1:**
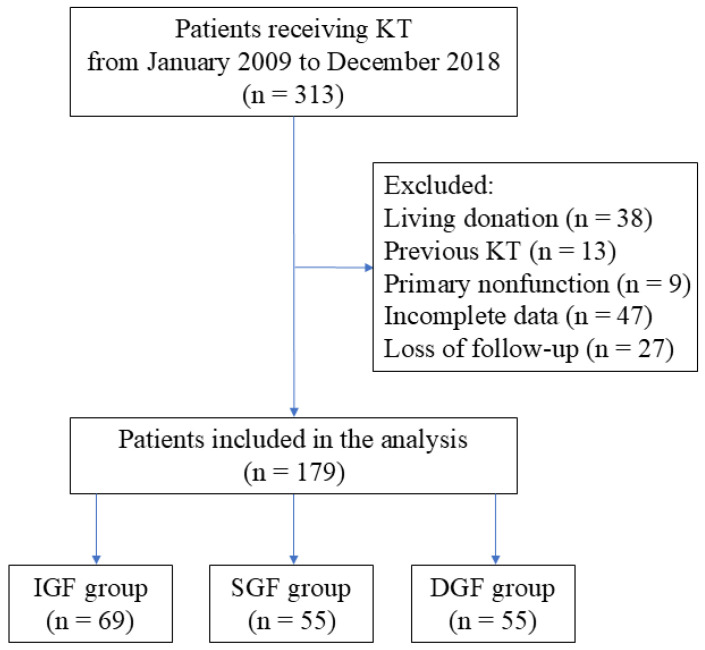
Flow diagram detailing the selection of patients included in the retrospective analysis. DGF, delayed graft function; IGF, immediate graft function; KT, kidney transplantation; SGF, slow graft function.

This retrospective analysis with consecutive adult patients who have undergone KT with allografts from deceased donation between January 2009 and December 2018 was conducted at Tri-Service General Hospital (TSGH), Taipei, Taiwan. The ethics committee of TSGH approved the study and waived the need for informed consent due to the retrospective design (TSGHIRB No: 1-108-05-047). Relevant clinical information was retrieved from the medical records and electronic database at TSGH. Patients enrolled for analysis were divided into the following 3 groups according to their early graft function: IGF, SGF and DGF. The exclusion criteria included age under 20 years, combined transplantation or KT after other solid organ transplantation, living-donor KT, previous KT, primary nonfunction and incomplete medical records. Ultimately, 134 cases were excluded from this analysis (Figure 1).

Patients who underwent living-donor KT and those with a history of previous KT were excluded to reduce clinical heterogeneity. Living-donor KT is typically associated with shorter ischemic times and superior graft quality compared with deceased-donor KT, which may contribute to improved early graft function and more stable perioperative conditions [18]. Similarly, recipients with prior KT may exhibit higher immunologic risk due to increased sensitization and alloimmune memory, which may adversely affect graft outcomes [19]. Inclusion of these populations could introduce substantial confounding and obscure the association between early postoperative BNP changes and graft outcomes. Therefore, this study focused on a relatively homogeneous cohort of first-time deceased-donor KT recipients to enhance internal validity.

### 2.2. Transplantation Procedure

All patients, except those receiving continuous peritoneal dialysis, underwent a dialysis session immediately before KT, targeting ultrafiltration to achieve a body weight 1–2 kg below the pretransplant dry weight. KT generally comprises two distinct surgical components: procurement of the donor kidney and preparation of the recipient for graft implantation. In this study, all procedures were performed using conventional surgical techniques for renal graft retrieval and subsequent graft transplantation.

Deceased-donor kidney procurement was performed through a midline laparotomy, followed by in situ aortic cannulation and perfusion with cold histidine–tryptophan–ketoglutarate (HTK) preservation solution to minimize ischemic injury. After aortic cross-clamping, both kidneys were retrieved *en bloc* with maximal vascular length. The renal arteries were excised with an aortic Carrel patch, and the renal veins were harvested with a cuff of the vena cava when appropriate. The ureters were dissected with an adequate margin of periureteric tissue to preserve their vascular supply. On the back table, the renal vessels and ureter were further dissected and trimmed, and excess perinephric tissue was removed. The grafts were flushed with cold HTK solution and stored on ice at approximately 4 °C until implantation.

Kidney implantation was performed using an extraperitoneal approach to the iliac fossa through a Rutherford–Morison incision. The external iliac vessels were exposed, and surrounding lymphatics were ligated. End-to-side vascular anastomoses were then completed sequentially, beginning with the renal vein to the external iliac vein, followed by the renal artery to the external iliac artery. During revascularization, continuous surface cooling with ice slush was applied to the graft. Upon completion of the anastomoses, the graft was revascularized, and adequate reperfusion was confirmed by immediate color change and turgor restoration. Urinary reconstruction was achieved using an extravesical ureteroneocystostomy via the Lich–Gregoir technique, ensuring a tension-free ureter with preservation of the periureteric tissue. A double-J stent was routinely inserted. After meticulous hemostasis, the graft was positioned in the iliac fossa without kinking of the vessels or ureter. Finally, a closed-suction drain was placed near the graft, and the surgical wound was closed in layers.

### 2.3. Immunosuppressive Therapy

Perioperative immunosuppressive regimens were administered in accordance with institutional practice and tailored to individual patient risk. Standard induction therapy with basiliximab (20 mg/day) was given on the day of surgery and again on postoperative day 4. For immunologically high-risk recipients, such as those with advanced donor age, a high number of human leukocyte antigen (HLA) mismatches, or an elevated panel reactive antibody (PRA) level [20], rabbit anti-human thymocyte globulin (r-ATG; 1.5 mg/kg intraoperatively, followed by same daily doses for 2 to 6 consecutive days) was used for induction immunosuppression.

The standard maintenance immunosuppression regimen at our institution consisted of a calcineurin inhibitor (either cyclosporine or tacrolimus), a purine synthesis inhibitor (mycophenolate mofetil), and a low-dose corticosteroid. Cyclosporine (6 mg/kg/day) or tacrolimus (0.3 mg/kg/day) was initiated in combination with mycophenolate mofetil (2000 mg/day), and drug levels were monitored and adjusted according to trough plasma concentrations. For cyclosporine, the target trough level was 150–300 ng/mL during the first 3 months, followed by tapering to maintain concentrations between 100 and 200 ng/mL; tacrolimus doses were titrated to achieve trough levels of 8–10 ng/mL during the first 3 months and 3–7 ng/mL thereafter [21]. Moreover, all patients received 500 mg of intravenous methylprednisolone on the day of surgery, followed by oral prednisolone at an initial dose of 20 mg.

### 2.4. Definitions of Graft Function

In the early postoperative period, enrolled patients were categorized into three groups according to graft function. DGF was defined as the need for dialysis within the first week after KT. SGF was defined as a sCr ≥ 3 mg/dL on postoperative day 5 without a dialysis requirement during the first postoperative week, whereas IGF was defined as a sCr < 3 mg/dL on postoperative day 5 [8].

### 2.5. Relevant Variables

Data related to the recipients, donors, and transplantation procedures were collected retrospectively. Recipient indicators included calendar period, age at the time of KT, gender, body mass index (BMI), comorbidity, primary cause of ESKD, modality and duration of renal replacement therapy, preoperative sCr level, number of HLA mismatches, and PRA titer. Donor details included age, gender, BMI, primary cause of death, donor criteria category, use of extracorporeal membrane oxygenation (ECMO), and terminal sCr level. Transplantation factors included cold and warm ischemia time, operation and anesthesia duration, anesthetic technique, need for intraoperative blood transfusion, and immunosuppressive agent for induction and maintenance. Cold ischemia time was defined as the interval from the initiation of cold perfusion to the removal of the graft from cold storage for implantation, while warm ischemia time was defined as the interval from the removal of the graft from cold storage to the restoration of blood flow after vascular anastomosis. In addition, serum BNP values of recipients measured before and after KT were recorded, and the percentage increase (dBNP) between preoperative BNP (preBNP) and postoperative BNP (postBNP) levels was calculated using the formula: dBNP = [(postBNP − preBNP)/preBNP] × 100%, where preBNP and postBNP refer to serum BNP levels measured after preoperative hemodialysis on the day of admission and within 24 h after KT, respectively.

Other clinical data included the length of intensive care unit (ICU) and total hospital stay, episode of acute rejection, estimated glomerular filtration rate (eGFR) values at different time points, incidence of graft failure at selected time intervals, and number of patients undergoing repeated KT. Graft failure, excluding patient death with a functioning graft, was defined as the development of irreversible graft injury requiring chronic dialysis or repeat KT.

### 2.6. Statistical Methods

The primary outcome was to evaluate the association between early postoperative BNP changes and long-term kidney allograft survival, and to investigate the discriminatory ability of BNP-derived parameters for allograft loss. The discriminatory performance of postBNP and dBNP for graft loss was assessed using receiver operating characteristic (ROC) curve analysis. The biomarker demonstrating superior discriminatory ability, as determined by the area under the curve (AUC), was subsequently analyzed to identify its optimal cutoff value. AUC values were interpreted as follows: 0.7–0.8, acceptable; 0.8–0.9, excellent; and >0.9, outstanding discrimination. Cutoff thresholds were identified using the Youden index, and patients were dichotomized into high- and low-value groups based on the superior biomarker for comparative analyses. Secondary outcomes included comparisons of recipient characteristics, donor details, transplantation-related factors, and postoperative indicators among the IGF, SGF, and DGF groups. In addition, the associations between these variables and graft loss were examined using Cox proportional hazards regression models. Univariate Cox proportional hazards regression was initially performed to identify variables associated with graft loss, and then variables with statistical significance in univariate analyses were entered into multivariate Cox models to determine factors independently associated with graft failure.

In the present study, continuous variables were presented as mean ± standard deviation (SD). They were analyzed using the independent *t*-test or the Mann–Whitney *U* test for comparisons between two groups, while one-way analysis of variance or the Kruskal–Wallis test was used for comparisons among three groups, according to the normality of data distribution. When statistically significant differences were identified among three groups, post hoc pairwise comparisons were performed using the Tukey’s honestly significant difference test for normally distributed variables and the Dunn–Bonferroni procedure for non-normally distributed variables. Normality of distribution was assessed using the Shapiro–Wilk test. Categorical variables were expressed as numbers with percentages and were compared using either the chi-square test or Fisher’s exact test, as appropriate. When overall group differences among three groups were statistically significant, post hoc pairwise comparisons were conducted using the Fisher’s exact test with Bonferroni correction. Kaplan–Meier survival curves were constructed to compare graft survival stratified by graft function categories (IGF, SGF, and DGF) and by ROC-derived biomarker groups. Survival time was defined as the interval from the date of KT to the date of graft loss or 31 December 2020, for censored patients. Differences in graft survival distributions were assessed using the log-rank test. A two-tailed *p* value < 0.05 was considered statistically significant. All statistical analyses were performed using SPSS Statistics (version 23.0; IBM SPSS Inc., Chicago, IL, USA).

## 3. Results

A total of 313 patients undergoing KT were initially screened for the study. After applying the exclusion criteria, 179 eligible patients remained in the final cohort for analysis. Among them, 69 patients were classified as having IGF, 55 as SGF, and 55 as DGF. The patient selection process and group distribution are illustrated in Figure 1.

### 3.1. Patient Demographics and Clinical Outcomes

Patient characteristics and posttransplant outcomes are presented in Table 1. Significant differences among the three graft function categories were observed in several recipient, donor, and transplantation-related characteristics, as well as posttransplant outcomes.

Recipients differed significantly in both PRA class I (IGF vs. SGF vs. DGF, 2.4 ± 11.9 vs. 6.9 ± 22.5 vs. 9.5 ± 25.0%; *p* = 0.026) and PRA class II (0.5 ± 4.1 vs. 7.6 ± 20.7 vs. 15.5 ± 32.9%; *p* = 0.002) levels among the three groups. Post hoc comparisons revealed significantly higher PRA class I levels in the DGF group than in the IGF group (*p* = 0.021), as well as significantly higher PRA class II levels in both the SGF (*p* = 0.042) and DGF (*p* = 0.003) groups compared with the IGF group. With respect to donor-related factors, ECMO use in donors differed significantly among the three graft function groups (2.9 vs. 10.9 vs. 16.4%; *p* = 0.036). Post hoc pairwise analyses indicated that the rate of ECMO utilization was significantly higher in the DGF group than in the IGF group (*p* = 0.033). In addition, cold ischemic time during KT differed significantly across the three groups (5.1 ± 1.8 vs. 5.9 ± 1.4 vs. 7.7 ± 1.4 h; *p* < 0.001). Subsequent post hoc comparisons showed that cold ischemic time was significantly longer in the DGF group than in both the IGF and SGF groups (both *p* < 0.001), whereas no significant difference was observed between the IGF and SGF groups (*p* = 0.096).

Regarding BNP-derived biomarkers, both postBNP levels (359.1 ± 70.5 vs. 376.7 ± 77.2 vs. 394.5 ± 51.9 pg/mL; *p* = 0.014) and dBNP ratios (17.3 ± 6.5 vs. 19.7 ± 5.7 vs. 22.4 ± 5.1%; *p* < 0.001) differed significantly among the three groups. Post hoc comparisons demonstrated that postBNP levels were significantly higher in the DGF group than in the IGF group (*p* = 0.012). Similarly, dBNP ratios were significantly elevated in the DGF group compared with both the IGF (*p* < 0.001) and SGF (*p* = 0.046) groups. No evidence of heart failure or other severe cardiovascular events was identified among the included patients.

As for posttransplant outcomes, significant differences were observed among the three graft function groups in ICU stay, total hospital stay, acute rejection rate, eGFR values at all evaluated time points, and the incidence of graft loss. Both the length of ICU stays (2.8 ± 0.9 vs. 3.0 ± 0.8 vs. 4.1 ± 2.4 days; *p* < 0.001) and total hospital stays (11.3 ± 3.5 vs. 11.5 ± 2.8 vs. 14.5 ± 4.8 days; *p* < 0.001) differed significantly among the groups, with post hoc analyses demonstrating significantly longer stays in the DGF group than in both the IGF and SGF groups (both *p* < 0.05). Acute rejection rates also differed significantly across the three groups (4.3 vs. 14.5 vs. 36.4%; *p* < 0.001), with significantly higher rates observed in the DGF group compared with the IGF (*p* < 0.001) and SGF (*p* = 0.045) groups. At each follow-up time point from 1 month to 5 years after KT, eGFR values differed significantly among the three groups. Post hoc analyses consistently demonstrated higher eGFR values in the IGF group than in both the SGF and DGF groups across all time points. Differences between the SGF and DGF groups were significant during the early postoperative period (≤6 months) but were no longer evident at later follow-up. The incidence of graft failure at 1, 3, and 5 years differed significantly among the groups, with higher failure rates observed in the DGF group than in the IGF group. Moreover, overall graft failure differed significantly across the three groups (20.3 vs. 38.2 vs. 40.0%; *p* = 0.031); however, post hoc pairwise comparisons showed only borderline significance between the DGF and IGF groups (*p* = 0.054).

### 3.2. Overall Kidney Graft Survival

**Figure 2:**
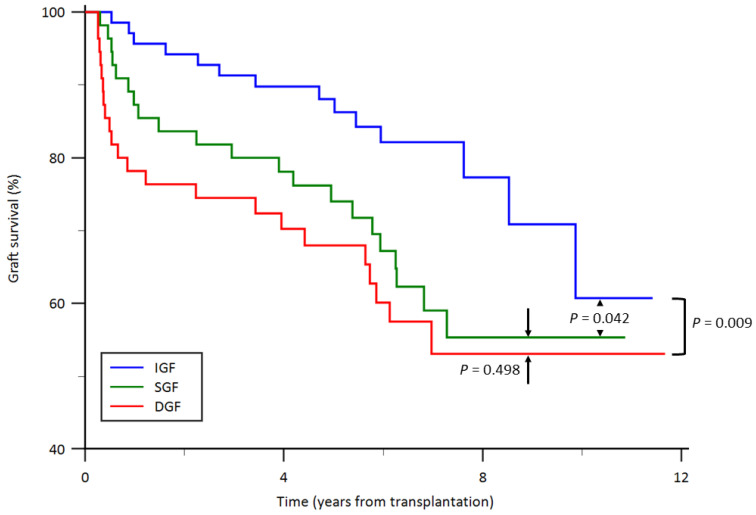
Graft survival curves from the date of kidney transplantation by graft function status. DGF, delayed graft function; IGF, immediate graft function; SGF, slow graft function.

Kaplan–Meier survival curves according to graft function status are illustrated in Figure 2. The median follow-up duration was 6.2 years in the IGF group, 6.3 years in the SGF group, and 5.2 years in the DGF group. No recipient deaths with a functioning graft occurred during the follow-up period. Kaplan–Meier analysis demonstrated a significant difference in overall kidney graft survival among the three graft function groups (*p* = 0.027). Pairwise log-rank comparisons showed that graft survival was significantly better in the IGF group than in both the DGF group (*p* = 0.009) and the SGF group (*p* = 0.042). In contrast, no significant difference in graft survival was observed between the SGF and DGF groups (*p* = 0.498). Consistent with these results, recipients in the DGF group exhibited poorer graft survival than those in the IGF group, characterized by earlier graft failure events, whereas the SGF group displayed an intermediate survival pattern.

### 3.3. Risks of Overall Kidney Graft Failure

The risk factors for overall kidney graft failure are shown in Table 2. In univariate analysis, patients undergoing KT with higher dBNP ratios had a significantly higher risk of graft loss compared with those with lower dBNP ratios (hazard ratio [HR], 1.17; 95% confidence interval [CI], 1.12–1.23; *p* < 0.001). After adjustment for calendar period, donor terminal sCr, postBNP level, graft function, and postoperative ICU and total hospital stays in the multivariate model, a higher dBNP ratio remained significantly associated with graft failure (HR, 1.16; 95% CI, 1.10–1.21; *p* < 0.001). Additionally, longer posttransplant ICU stay was independently associated with an increased risk of graft loss in the multivariate analysis (*p* = 0.020).

### 3.4. Discriminatory Performance of BNP-Derived Biomarkers for Graft Failure

**Figure 3:**
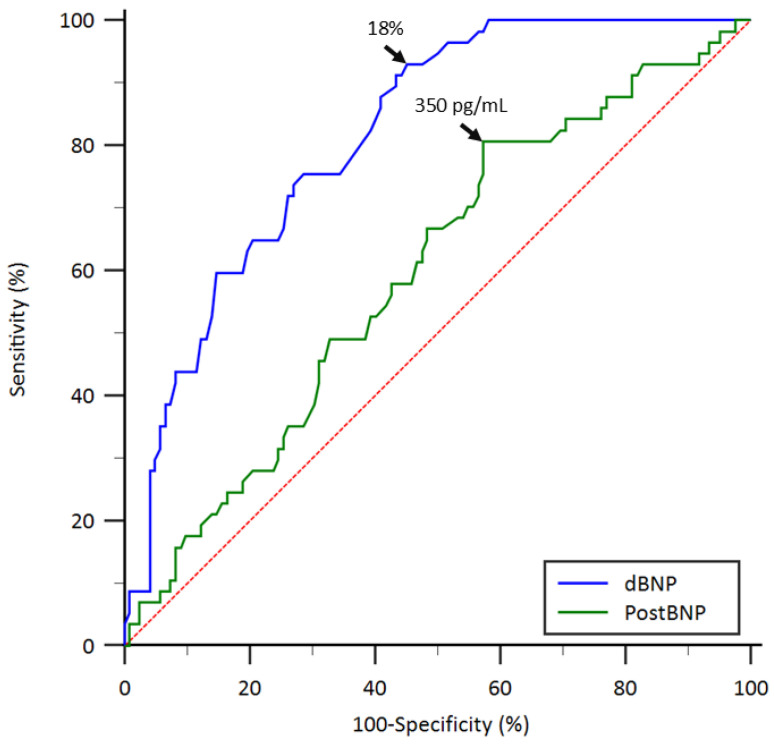
Receiver operating characteristic curves comparing the discriminatory performance of dBNP and postBNP for predicting graft failure after kidney transplantation. dBNP, percentage change in serum brain natriuretic peptide levels before and after kidney transplantation; postBNP, posttransplant serum brain natriuretic peptide level.

ROC analyses of BNP-derived biomarkers to identify the optimal parameter for predicting graft failure are presented in Figure 3. The Youden index reached its maximum at a postBNP value of 350 pg/mL, yielding an AUC of 0.596 (95% CI, 0.52–0.67; *p* = 0.032), with a sensitivity of 80.7% and a specificity of 42.6%. For the dBNP ratio, the Youden index peaked at 18%, with an AUC of 0.815 (95% CI, 0.75–0.87; *p* < 0.001), corresponding to a sensitivity of 93.0% and a specificity of 54.9%. Comparison of the ROC curves demonstrated a significant difference between the dBNP ratio and postBNP level (*p* < 0.001). Based on the identified cutoff value of the dBNP ratio, participants were categorized as having a higher (≥ 18%) or lower (<18%) dBNP ratio.

### 3.5. Patient Characteristics and Posttransplant Outcomes by Categorized dBNP Ratio

Patient characteristics and posttransplant outcomes stratified by the dBNP ratio (≥18% vs. <18%) are summarized in Table 3. Recipients with higher dBNP ratios were more likely to have longer cold ischemic times, unfavorable early graft function, and lower eGFR values during follow-up. In addition, higher dBNP ratios were associated with an increased incidence of graft failure. These findings should be interpreted as exploratory and descriptive, given the limitations of the cutoff-based analysis.

**Figure 4:**
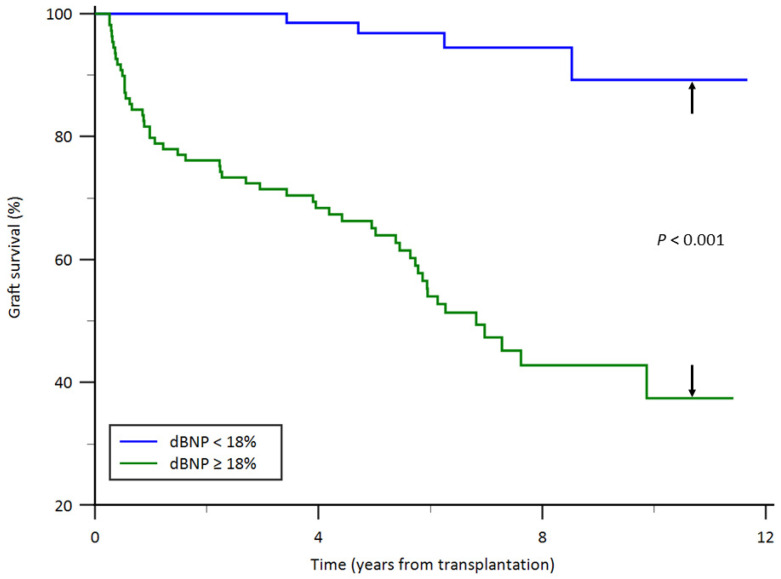
Graft survival curves from the date of kidney transplantation by dBNP ratio. dBNP, percentage increase in serum brain natriuretic peptide levels before and after kidney transplantation.

Kaplan–Meier survival curves according to dBNP ratio are illustrated in Figure 4. Kaplan–Meier analysis demonstrated a significant difference in overall kidney graft survival between the two dBNP ratio groups (*p* < 0.001), with higher graft survival observed in recipients with lower dBNP ratios compared with those with higher dBNP ratios.

## 4. Discussion

This study evaluated potential biomarkers for kidney allograft survival in patients undergoing KT and demonstrated that early postoperative changes in BNP levels, expressed as the dBNP ratio (percentage increase from pretransplant to postoperative day 1), were significantly associated with posttransplant graft outcomes. Recipients with higher dBNP ratios exhibited poorer early graft function, lower eGFR values at multiple follow-up time points, and a higher incidence of graft failure. Notably, the dBNP ratio demonstrated superior discriminatory performance compared with postBNP alone, with an exploratory cutoff of approximately 18%. Given that long-term graft survival remains a major challenge despite improvements in short-term outcomes after KT [1,4], the present findings may provide additional insight into the association between early postoperative BNP dynamics and graft outcomes, which contribute to understanding early posttransplant physiological changes.

BNP is synthesized and released primarily by ventricular cardiomyocytes in response to myocardial wall stress and volume expansion, and its circulating concentration indicates complex interactions among cardiac function, intravascular volume status, and renal clearance [22]. A prior study showed that serum BNP levels are elevated in patients with chronic kidney disease before KT and typically decline with successful allograft function; in contrast, a pronounced rebound or increase in BNP levels immediately after KT may indicate impaired early graft function [23]. Previous studies in dialysis populations have also demonstrated that serum BNP levels correlate with hydration status and objective measures of volume overload [15,16,17]. Collectively, BNP may reflect underlying cardiorenal physiology and volume status in the early posttransplant period; however, its role as a clinical biomarker for graft outcomes remains to be established.

In our study, although elevated postBNP levels were correlated with poorer early allograft function and adverse outcomes, their prognostic utility appeared limited, likely due to the influence of preexisting cardiorenal conditions unrelated to graft performance in KT recipients. In contrast to static biomarkers measured at a single time point, the dBNP ratio captures dynamic physiological responses to KT by integrating both pretransplant baseline levels and immediate postoperative changes. This approach is particularly relevant in patients with ESKD, in whom baseline BNP levels are frequently elevated because of chronic volume overload, reduced renal clearance, and a high burden of cardiac comorbidities [14]. These underlying conditions may confound the interpretation of isolated BNP measurements. By emphasizing relative changes from the baseline, the dBNP ratio may better reflect perioperative physiological stress and early graft-related volume regulation. However, these findings should be considered hypothesis-generating, and further prospective studies are required to determine whether BNP-derived parameters have clinical utility in predicting or guiding graft-related outcomes after KT.

Consistent with these observations, recipients with higher dBNP ratios in the present study were more likely to have longer cold ischemic times, higher rates of DGF, and persistent renal dysfunction during follow-up, suggesting that the dBNP ratio may indicate perioperative physiological stress and early graft dysfunction. Several prognostically relevant variables, including PRA levels, donor ECMO use, cold ischemia time, acute rejection, ICU stay, and early graft function, differed between groups and may be associated with both BNP dynamics and graft outcomes. These interrelated factors indicate that the observed association between the dBNP ratio and graft survival may be influenced by residual confounding and shared underlying pathophysiological pathways. Accordingly, the dBNP ratio should be interpreted as an integrated marker of perioperative severity and early graft function rather than a definitive clinical decision-making tool, given the lack of external validation. Of note, under our institutional protocol, most recipients underwent dialysis immediately before KT; therefore, the dBNP ratio in this study was generally expressed as a positive percentage change. This methodological difference from a prior study focusing on absolute BNP values at specific postoperative time points [23] may partly explain discrepancies in reported BNP dynamics between studies.

Early graft function status, conventionally classified as IGF, SGF, and DGF, is a key determinant of both short- and long-term outcomes after KT [24,25,26]. DGF has been consistently associated with inferior graft survival, higher rates of acute rejection, and increased healthcare utilization [5,7]. In contrast, the clinical significance of SGF remains controversial, largely due to heterogeneity in its definitions and timing of assessment. Although SGF has been proposed as an intermediate phenotype between IGF and DGF [25], emerging evidence suggests that its long-term risk of graft loss may be comparable to that of DGF [24,26]. In our cohort, recipients with poorer early graft function, including SGF and DGF, exhibited significantly worse graft survival, consistent with previous reports. Importantly, conventional classification of early graft function requires several postoperative days to be established, limiting its utility for early risk stratification; conversely, the dBNP ratio measured within 24 h postoperatively provided prognostic information that paralleled, and may precede, traditional early graft function categories.

Cold ischemia is a well-established contributor to ischemia–reperfusion injury, impairing early tubular function and delaying recovery of natriuresis and fluid regulation, thereby increasing the risk of DGF and subsequent graft failure [27]. In our cohort, recipients with higher dBNP ratios had longer cold ischemic times and poorer early graft function. Although cold ischemic time was not independently associated with long-term allograft loss, it appeared related to the incidence of DGF, as reported in prior studies [5,6,7,10]. Thus, efforts to minimize cold ischemic time may help reduce the risk of DGF. In addition to the dBNP ratio, prolonged ICU stay was independently associated with inferior graft outcomes, likely indicating greater perioperative illness severity and early postoperative complications, consistent with findings from a previous report [7].

Our study has several limitations. First, as a retrospective cohort study conducted at a single medical center, there was an inherent risk of selection bias and residual confounding, and the relatively modest sample size may limit the applicability of our findings. Although multivariate analyses were performed to adjust for multiple variables, unmeasured confounders may still have influenced the observed associations. Second, detailed data on perioperative fluid therapy, hemodynamic support, and volume status were not consistently available in this study. Given that BNP levels are influenced by hemodynamic conditions and volume-related factors, these unmeasured variables may have contributed to residual confounding, and the association between BNP-derived parameters and graft outcomes should therefore be interpreted with caution. Third, several biomarker-specific limitations should be considered. N-terminal pro-BNP, which has a longer half-life and greater analytical stability than BNP, was not assessed in this study. BNP levels may also be influenced by subclinical cardiovascular comorbidities, such as left ventricular hypertrophy or diastolic dysfunction, which are common in patients with ESKD and were not fully characterized in this cohort. Furthermore, both the biological and analytical variability of BNP, including fluctuations related to volume status, dialysis timing, and assay differences, may affect the precision and reproducibility of BNP-based measurements. Fourth, this study included only recipients of deceased-donor KT and first-time transplant recipients; therefore, the findings may not be directly generalizable to other transplant populations. Finally, the findings were derived from a single-center retrospective cohort and have not undergone external validation. Therefore, the proposed associations and cutoff values should be considered exploratory and hypothesis-generating, and further prospective, multicenter studies are required to confirm their clinical applicability. Despite these limitations, the present findings may provide additional insight into early posttransplant physiological processes associated with allograft survival after KT in patients with ESKD.

## 5. Conclusions

This study suggests that the dBNP ratio reflects early postoperative cardiorenal stress and volume-related burden that may adversely affect graft recovery and long-term function. Given its simplicity, objectivity, and availability in routine clinical practice, the dBNP ratio may represent a useful indicator associated with early postoperative physiological changes and graft outcomes. However, its clinical applicability requires further prospective, multicenter studies to validate these findings and to determine whether dBNP-guided interventions can improve long-term graft outcomes after KT.

## Figures and Tables

**Table 1:** Demographic characteristics and clinical outcomes of patients undergoing kidney transplantation with different graft function.

Variables	Groups			Total (*n* = 179)	*p* Value
	IGF (*n* = 69)	SGF (*n* = 55)	DGF (*n* = 55)		
**Recipient**					
Calendar period, *n* (%)					0.107
2009–2013	25 (36.2)	30 (54.5)	27 (49.1)	82 (45.8)	
2014–2018	44 (63.8)	25 (45.5)	28 (50.9)	97 (54.2)	
Age (years), mean (SD)	41.8 (11.8)	42.6 (12.1)	47.0 (12.0)	43.7 (12.1)	0.071
Sex, *n* (%)					0.110
Male	34 (49.3)	33 (60.0)	22 (40.0)	89 (49.7)	
Female	35 (50.7)	22 (40.0)	33 (60.0)	90 (50.3)	
BMI (kg/m^2^), mean (SD)	22.2 (2.7)	23.3 (3.7)	23.4 (3.5)	22.9 (3.3)	0.088
Comorbidities, *n* (%)					
Hypertension	63 (91.3)	48 (87.3)	50 (90.9)	161 (89.9)	0.729
Diabetes mellitus	14 (20.3)	8 (14.5)	7 (12.7)	29 (16.2)	0.484
Cardiac disease	3 (4.3)	5 (9.1)	4 (7.3)	12 (6.7)	0.565
Hepatic disease	6 (8.7)	10 (18.2)	11 (20.0)	27 (15.1)	0.161
Rheumatic disease	6 (8.7)	3 (5.5)	3 (5.5)	12 (6.7)	0.700
Primary cause of renal failure, *n* (%)					0.731
Diabetic nephropathy	8 (11.6)	6 (10.9)	6 (10.9)	20 (11.2)	
Hypertensive nephropathy	14 (20.3)	12 (21.8)	15 (27.3)	41 (22.9)	
Glomerulonephritis	21 (30.4)	18 (32.7)	11 (20.0)	50 (27.9)	
Drug-induced nephropathy	6 (8.7)	5 (9.1)	6 (10.9)	17 (9.5)	
Lupus nephritis	6 (8.7)	3 (5.5)	2 (3.6)	11 (6.2)	
Others	5 (7.3)	7 (12.7)	4 (7.3)	16 (8.9)	
Unknown	9 (13.0)	4 (7.3)	11 (20.0)	24 (13.4)	
Type of dialysis, *n* (%)					0.915
Hemodialysis	28 (40.6)	23 (41.8)	25 (45.5)	76 (42.5)	
Peritoneal dialysis	33 (47.8)	28 (50.9)	25 (45.5)	86 (48.0)	
Combined therapy	8 (11.6)	4 (7.3)	5 (9.0)	17 (9.5)	
Pretransplant dialysis duration (months), mean (SD)	48.6 (43.7)	55.8 (43.4)	56.4 (37.0)	53.2 (41.6)	0.150
Pretransplant serum creatinine (mg/dL), mean (SD)	11.4 (3.8)	11.6 (4.2)	11.9 (4.1)	11.6 (4.0)	0.900
HLA mismatch, *n* (%)					0.072
0	4 (5.8)	4 (7.3)	0 (0.0)	8 (4.5)	
1–3	42 (60.9)	36 (65.4)	28 (50.9)	106 (59.2)	
4–6	23 (33.3)	15 (27.3)	27 (49.1)	65 (36.3)	
PRA (%), mean (SD)					
Class I	2.4 (11.9)	6.9 (22.5)	9.5 (25.0)	6.0 (20.2)	0.026
Class II	0.5 (4.1)	7.6 (20.7)	15.5 (32.9)	7.3 (22.4)	0.002
**Donor**					
Age (years), mean (SD)	39.7 (12.2)	40.8 (15.0)	45.1 (13.6)	41.7 (13.7)	0.073
Sex, *n* (%)					0.153
Male	55 (79.7)	36 (65.5)	37 (67.3)	128 (71.5)	
Female	14 (20.3)	19 (34.5)	18 (32.7)	51 (28.5)	
BMI (kg/m^2^), mean (SD)	21.9 (3.1)	21.9 (4.3)	22.9 (4.0)	22.2 (3.8)	0.308
Primary cause of death, *n* (%)					0.988
Trauma	28 (40.6)	21 (38.2)	20 (36.4)	69 (38.5)	
Cerebrovascular accident	32 (46.4)	25 (45.5)	26 (47.3)	83 (46.4)	
Anoxia	8 (11.6)	7 (12.7)	7 (12.7)	22 (12.3)	
Others	1 (1.4)	2 (3.6)	2 (3.6)	5 (2.8)	
Donor criteria, *n* (%)					0.103
SCD	50 (72.5)	37 (67.3)	32 (58.2)	119 (66.5)	
ECD	19 (27.5)	16 (29.1)	18 (32.7)	53 (29.6)	
DCD	0 (0.0)	2 (3.6)	5 (9.1)	7 (3.9)	
Use of ECMO, *n* (%)	2 (2.9)	6 (10.9)	9 (16.4)	17 (9.5)	0.036
Terminal serum creatinine (mg/dL), mean (SD)	1.0 (0.6)	1.2 (0.8)	1.4 (0.9)	1.2 (0.8)	0.131
**Transplantation**					
Cold ischemic time (hours), mean (SD)	5.1 (1.8)	5.9 (1.4)	7.7 (1.4)	6.2 (1.9)	<0.001
Warm ischemic time (minutes), mean (SD)	27.0 (11.1)	27.7 (11.5)	29.1 (12.3)	27.9 (11.6)	0.543
Operation time (minutes), mean (SD)	242.0 (51.9)	255.2 (63.5)	257.0 (57.6)	250.7 (57.5)	0.478
Anesthesia time (minutes), mean (SD)	295.0 (50.7)	309.5 (64.7)	310.4 (56.1)	304.2 (57.1)	0.499
Anesthetic technique, *n* (%)					0.387
INHA	55 (79.7)	38 (69.1)	42 (76.4)	135 (75.4)	
TIVA	14 (20.3)	17 (30.9)	13 (23.6)	44 (24.6)	
Intraoperative transfusion, *n* (%)	16 (23.2)	21 (38.2)	17 (30.9)	54 (30.2)	0.193
Immunotherapy induction, *n* (%)					0.052
Basiliximab	43 (62.3)	33 (60.0)	23 (41.8)	99 (55.3)	
r-ATG	26 (37.7)	22 (40.0)	32 (58.2)	80 (44.7)	
Calcineurin inhibitor, *n* (%)					0.670
Cyclosporine	14 (20.3)	11 (20.0)	8 (14.5)	33 (18.4)	
Tacrolimus	55 (79.7)	44 (80.0)	47 (85.5)	146 (81.6)	
**BNP Level**					
PreBNP (pg/mL), mean (SD)	306.7 (60.9)	314.7 (62.3)	322.7 (43.6)	314.1 (56.7)	0.308
PostBNP (pg/mL), mean (SD)	359.1 (70.5)	376.7 (77.2)	394.5 (51.9)	375.4 (68.8)	0.014
dBNP (%), mean (SD)	17.3 (6.5)	19.7 (5.7)	22.4 (5.1)	19.6 (6.2)	<0.001
**Posttransplant Outcome**					
Hospitalization (days), mean (SD)					
ICU stay	2.8 (0.9)	3.0 (0.8)	4.1 (2.4)	3.3 (1.6)	<0.001
Hospital stay	11.3 (3.5)	11.5 (2.8)	14.5 (4.8)	12.4 (4.0)	<0.001
Acute rejection, *n* (%)	3 (4.3)	8 (14.5)	20 (36.4)	31 (17.3)	<0.001
eGFR (mL/min/1.73 m^2^), mean (SD)					
1-month	62.0 (23.0)	44.9 (24.3)	33.3 (18.2)	47.9 (25.0)	<0.001
3-month	64.6 (23.4)	50.8 (25.2)	37.4 (23.4)	52.0 (26.4)	<0.001
6-month	66.7 (23.2)	56.6 (32.3)	40.5 (23.8)	55.6 (28.5)	<0.001
12-month	63.4 (27.8)	50.6 (26.1)	42.6 (25.4)	53.1 (27.9)	<0.001
36-month	59.9 (27.5)	44.3 (28.2)	40.1 (24.6)	49.0 (28.1)	<0.001
60-month	59.8 (30.4)	45.9 (30.4)	40.9 (27.3)	49.7 (30.5)	0.002
Graft loss, *n* (%)					
1-year	3 (4.3)	7 (12.7)	12 (21.8)	22 (12.3)	0.013
3-year	6 (8.7)	11 (20.0)	14 (25.5)	31 (17.3)	0.041
5-year	8 (11.6)	14 (25.5)	17 (30.9)	39 (21.8)	0.026
Total	14 (20.3)	21 (38.2)	22 (40.0)	57 (31.8)	0.031
Repeated transplantation, *n* (%)	1 (1.4)	2 (3.6)	3 (5.5)	6 (3.4)	0.464

Notes: dBNP = [(postoperative BNP − preoperative BNP)/preoperative BNP] × 100%. BMI, body mass index; BNP, brain natriuretic peptide; DCD, donor after cardiac death; DGF, delayed graft function; ECD, expanded criteria donor; ECMO, extracorporeal membrane oxygenation; eGFR, estimated glomerular filtration rate; HLA, human leukocyte antigen; ICU, intensive care unit; IGF, immediate graft function; INHA, inhalational anesthesia; PRA, panel reactive antibody; Pre/PostBNP, preoperative/postoperative brain natriuretic peptide; r-ATG, rabbit anti-human thymocyte globulin; SCD, standard criteria donor; SD, standard deviation; SGF, slow graft function; TIVA, total intravenous anesthesia.

**Table 2:** Cox proportional hazards regression for graft loss: univariate and multivariate models for kidney transplantation patients.

Variables	Univariate		Multivariate	
	HR (95% CI)	*p* Value	HR (95% CI)	*p* Value
**Recipient**				
Calendar period, 2014–2018 (ref: 2009–2013)	0.53 (0.30–0.94)	0.029	0.74 (0.40–1.36)	0.334
Age (years)	1.01 (0.99–1.03)	0.519		
Sex, female (ref: male)	0.97 (0.58–1.63)	0.903		
BMI (kg/m^2^)	1.04 (0.96–1.13)	0.307		
Comorbidities (ref: none)				
Hypertension	0.79 (0.34–1.84)	0.578		
Diabetes mellitus	0.79 (0.37–1.67)	0.537		
Cardiac disease	1.00 (0.36–2.77)	0.999		
Hepatic disease	1.72 (0.89–3.33)	0.107		
Rheumatic disease	1.25 (0.50–3.14)	0.634		
Primary cause of renal failure (ref: diabetic nephropathy)				
Hypertensive nephropathy	1.17 (0.48–2.86)	0.736		
Glomerulonephritis	0.87 (0.35–2.17)	0.770		
Drug-induced nephropathy	1.15 (0.39–3.43)	0.801		
Lupus nephritis	1.22 (0.39–3.85)	0.735		
Others	0.52 (0.13–2.02)	0.345		
Unknown	0.80 (0.28–2.29)	0.683		
Type of dialysis (ref: hemodialysis)				
Peritoneal dialysis	1.18 (0.69–2.03)	0.545		
Combined therapy	0.53 (0.16–1.77)	0.304		
Pretransplant dialysis duration (months)	1.00 (0.99–1.01)	0.759		
Pretransplant serum creatinine (mg/dL)	1.04 (0.98–1.11)	0.234		
HLA mismatch (ref: 0)				
1–3	3.17 (0.44–23.2)	0.255		
4–6	2.52 (0.34–18.8)	0.368		
PRA (%)				
Class I	0.97 (0.95–1.00)	0.085		
Class II	0.99 (0.97–1.01)	0.157		
**Donor**				
Age (years)	1.01 (0.99–1.03)	0.162		
Sex, female (ref: male)	1.11 (0.63–1.95)	0.729		
BMI (kg/m^2^)	0.99 (0.92–1.05)	0.662		
Primary cause of death (ref: trauma)				
Cerebrovascular accident	0.98 (0.55–1.76)	0.947		
Anoxia	1.29 (0.59–2.83)	0.518		
Others	2.85 (0.85–9.61)	0.091		
Donor criteria (ref: SCD)				
ECD	0.92 (0.51–1.65)	0.774		
DCD	2.10 (0.75–5.90)	0.160		
Use of ECMO (ref: none)	1.98 (0.97–4.04)	0.061		
Terminal serum creatinine (mg/dL)	1.52 (1.13–2.06)	0.006	1.17 (0.81–1.68)	0.416
**Transplantation**				
Cold ischemic time (hours)	1.08 (0.94–1.24)	0.295		
Warm ischemic time (minutes)	0.99 (0.97–1.01)	0.377		
Operation time (minutes)	1.00 (1.00–1.01)	0.483		
Anesthesia time (minutes)	1.00 (1.00–1.01)	0.446		
Anesthetic technique, TIVA (ref: INHA)	1.17 (0.66–2.09)	0.594		
Intraoperative transfusion (ref: none)	1.18 (0.68–2.07)	0.559		
Immunotherapy induction, r-ATG (ref: basiliximab)	0.65 (0.38–1.12)	0.121		
Calcineurin inhibitor, tacrolimus (ref: cyclosporine)	0.95 (0.48–1.89)	0.889		
**BNP Level**				
PreBNP (pg/mL)	1.00 (1.00–1.01)	0.877		
PostBNP (pg/mL)	1.00 (1.00–1.01)	0.044	1.00 (1.00–1.01)	0.302
dBNP (%)	1.17 (1.12–1.23)	<0.001	1.16 (1.10–1.21)	<0.001
**Posttransplant Outcome**				
Graft function (ref: IGF)				
SGF	1.98 (1.01–3.89)	0.048	1.34 (0.66–2.75)	0.422
DGF	2.40 (1.23–4.70)	0.010	1.04 (0.50–2.16)	0.921
Hospitalization (days)				
ICU stay	1.35 (1.21–1.51)	<0.001	1.22 (1.03–1.44)	0.020
Hospital stay	1.13 (1.08–1.19)	<0.001	1.05 (0.98–1.12)	0.207
Acute rejection (ref: none)	0.65 (0.30–1.44)	0.287		

Notes: Hazard ratios in the multivariate analyses were adjusted by those variables having significance in the univariate analyses. dBNP = [(postoperative BNP − preoperative BNP)/preoperative BNP] × 100%. BMI, body mass index; BNP, brain natriuretic peptide; DCD, donor after cardiac death; DGF, delayed graft function; ECD, expanded criteria donor; ECMO, extracorporeal membrane oxygenation; HLA, human leukocyte antigen; HR, hazard ratio; ICU, intensive care unit; IGF, immediate graft function; INHA, inhalational anesthesia; PRA, panel reactive antibody; Pre/PostBNP, preoperative/postoperative brain natriuretic peptide; r-ATG, rabbit anti-human thymocyte globulin; SCD, standard criteria donor; SGF, slow graft function; TIVA, total intravenous anesthesia.

**Table 3:** Demographic characteristics and clinical outcomes of patients undergoing kidney transplantation with different dBNP ratios.

Variables	Groups		*p* Value
	dBNP ≥ 18% (*n* = 109)	dBNP < 18% (*n* = 70)	
**Recipient**			
Calendar period, *n* (%)			0.127
2009–2013	55 (50.5)	27 (38.6)	
2014–2018	54 (49.5)	43 (61.4)	
Age (years), mean (SD)	44.0 (12.1)	43.1 (12.2)	0.557
Sex, *n* (%)			0.879
Male	55 (50.5)	34 (48.6)	
Female	54 (49.5)	36 (51.4)	
BMI (kg/m^2^), mean (SD)	23.1 (3.1)	22.6 (3.7)	0.321
Comorbidities, *n* (%)			
Hypertension	98 (89.9)	63 (90.0)	1.000
Diabetes mellitus	19 (17.4)	10 (14.3)	0.679
Cardiac disease	8 (7.3)	4 (5.7)	0.767
Hepatic disease	18 (16.5)	9 (12.9)	0.669
Rheumatic disease	10 (9.2)	2 (2.9)	0.130
Primary cause of renal failure, *n* (%)			0.429
Diabetic nephropathy	15 (13.8)	5 (7.1)	
Hypertensive nephropathy	26 (23.8)	15 (21.4)	
Glomerulonephritis	29 (26.6)	21 (30.0)	
Drug-induced nephropathy	8 (7.3)	9 (12.9)	
Lupus nephritis	9 (8.3)	2 (2.9)	
Others	9 (8.3)	7 (10.0)	
Unknown	13 (11.9)	11 (15.7)	
Type of dialysis, *n* (%)			0.327
Hemodialysis	51 (46.8)	25 (35.7)	
Peritoneal dialysis	49 (44.9)	37 (52.9)	
Combined therapy	9 (8.3)	8 (11.4)	
Pretransplant dialysis duration (months), mean (SD)	56.4 (41.4)	48.3 (41.7)	0.064
Pretransplant serum creatinine (mg/dL), mean (SD)	11.5 (3.9)	11.9 (4.2)	0.307
HLA mismatch, *n* (%)			0.811
0	4 (3.7)	4 (5.7)	
1–3	65 (59.6)	41 (58.6)	
4–6	40 (36.7)	25 (35.7)	
PRA (%), mean (SD)			
Class I	5.4 (18.1)	6.9 (23.1)	0.768
Class II	7.5 (22.8)	7.0 (22.1)	0.597
**Donor**			
Age (years), mean (SD)	42.2 (13.8)	40.8 (13.6)	0.546
Sex, *n* (%)			0.397
Male	75 (68.8)	53 (75.7)	
Female	34 (31.2)	17 (24.3)	
BMI (kg/m^2^), mean (SD)	22.0 (3.5)	22.5 (4.2)	0.728
Primary cause of death, *n* (%)			0.903
Trauma	41 (37.6)	28 (40.0)	
Cerebrovascular accident	50 (45.9)	33 (47.1)	
Anoxia	15 (13.8)	7 (10.0)	
Others	3 (2.7)	2 (2.9)	
Donor criteria, *n* (%)			0.793
SCD	73 (67.0)	46 (65.7)	
ECD	31 (28.4)	22 (31.4)	
DCD	5 (4.6)	2 (2.9)	
Use of ECMO, *n* (%)	13 (11.9)	4 (5.7)	0.199
Terminal serum creatinine (mg/dL), mean (SD)	1.3 (0.8)	1.1 (0.7)	0.191
**Transplantation**			
Cold ischemic time (hours), mean (SD)	6.5 (1.8)	5.6 (1.9)	0.007
Warm ischemic time (minutes), mean (SD)	28.2 (12.4)	27.7 (11.1)	0.758
Operation time (minutes), mean (SD)	249.5 (53.1)	252.5 (64.0)	0.975
Anesthesia time (minutes), mean (SD)	303.5 (53.0)	305.4 (63.3)	0.981
Anesthetic technique, *n* (%)			0.480
INHA	80 (73.4)	55 (78.6)	
TIVA	29 (26.6)	15 (21.4)	
Intraoperative transfusion, *n* (%)	31 (28.4)	23 (32.9)	0.617
Immunotherapy induction, *n* (%)			0.759
Basiliximab	59 (54.1)	40 (57.1)	
r-ATG	50 (45.9)	30 (42.9)	
Calcineurin inhibitor, *n* (%)			0.117
Cyclosporine	16 (14.7)	17 (24.3)	
Tacrolimus	93 (85.3)	53 (75.7)	
**Posttransplant Outcome**			
Hospitalization (days), mean (SD)			
ICU stay	3.5 (1.9)	3.0 (1.0)	0.109
Hospital stay	12.9 (4.5)	11.5 (2.8)	0.096
Graft function			<0.001
IGF	30 (27.5)	39 (55.7)	
SGF	36 (33.0)	19 (27.1)	
DGF	43 (39.5)	12 (17.2)	
Acute rejection, *n* (%)	21 (19.3)	10 (14.3)	0.426
eGFR (mL/min/1.73 m^2^), mean (SD)			
1-month	43.9 (25.4)	54.1 (23.4)	0.004
3-month	47.8 (28.3)	58.7 (21.6)	0.003
6-month	50.9 (31.9)	62.8 (20.5)	<0.001
12-month	46.3 (29.1)	63.7 (22.0)	<0.001
36-month	41.1 (28.7)	61.4 (22.3)	<0.001
60-month	40.6 (30.7)	63.9 (24.1)	<0.001
Graft loss, *n* (%)			
1-year	22 (20.2)	0 (0.0)	<0.001
3-year	31 (28.4)	0 (0.0)	<0.001
5-year	37 (33.9)	2 (2.9)	<0.001
Total	53 (48.6)	4 (5.7)	<0.001
Repeated transplantation, *n* (%)	6 (5.5)	0 (0.0)	0.083

Notes: dBNP = [(postoperative BNP − preoperative BNP)/preoperative BNP] × 100%. BMI, body mass index; BNP, brain natriuretic peptide; DCD, donor after cardiac death; DGF, delayed graft function; ECD, expanded criteria donor; ECMO, extracorporeal membrane oxygenation; eGFR, estimated glomerular filtration rate; HLA, human leukocyte antigen; ICU, intensive care unit; IGF, immediate graft function; INHA, inhalational anesthesia; PRA, panel reactive antibody; r-ATG, rabbit anti-human thymocyte globulin; SCD, standard criteria donor; SD, standard deviation; SGF, slow graft function; TIVA, total intravenous anesthesia.

## Data Availability

The data analyzed in this study are available from the corresponding author on reasonable request.
